# Primary healthcare seeking behaviour of low-income patients across the public and private health sectors in South Africa

**DOI:** 10.1186/s12889-021-11678-9

**Published:** 2021-09-09

**Authors:** Kerensa Govender, Sarah Girdwood, Daniel Letswalo, Lawrence Long, G. Meyer-Rath, J. Miot

**Affiliations:** 1 Department of Internal Medicine, Faculty of Health Sciences, University of the Witwatersrand, Johannesburg, South Africa; 2 Health Economics and Epidemiology Research Office (HE2RO), Wits Health Consortium, University of the Witwatersrand, Johannesburg, South Africa; 3 Department of Global Health, Boston University School of Public Health, Boston, Mass USA

**Keywords:** Primary healthcare, Private sector, NHI, Preferences, Utilisation, Health care seeking behaviour

## Abstract

**Background:**

The proposed National Health Insurance (NHI) system aims to re-engineer primary healthcare (PHC) in South Africa, envisioning both private sector providers and public sector clinics as independent contracting units to the NHI Fund. In 2017, 16% of the South African population had private medical insurance and predominately utilised private providers. However, it is estimated that up to 28% of the population access private PHC services, with a meaningful segment of the low-income, uninsured population paying for these services out-of-pocket. The study objective was to characterise the health seeking behaviour of low-income, patients accessing PHC services in both the public and private sectors, patient movement between sectors, and factors influencing their facility choice.

**Methods:**

We conducted once-off patient interviews on a random sample of 153 patients at 7 private PHC providers (primarily providing services to the low-income mostly uninsured patient population) and their matched public PHC clinic (7 facilities).

**Results:**

The majority of participants were economically active (96/153, 63%), 139/153 (91%) did not have health insurance, and 104/153 (68%) earned up to $621/month. A multiple response question found affordability (67%) and convenience (60%) were ranked as the most important reasons for choosing to usually access care at public clinics (48%); whilst convenience (71%) and quality of care (59%) were key reasons for choosing the private sector (32%). There is movement between sectors: 23/76 (30%) of those interviewed at a private facility and 8/77 (10%) of those interviewed at a public facility indicated usually accessing PHC services at a mix of private and public facilities. Results indicate cycling between the private and public sectors with different factors influencing facility choice.

**Conclusions:**

It is imperative to understand the potential impact on where PHC services are accessed once affordability is mitigated through the NHI as this has implications on planning and contracting of services under the NHI.

## Background

National health insurance (NHI) has emerged as a key component of existing health financing reforms in middle and low income countries and is critical to the attainment of universal health coverage (UHC) [1]. In South Africa the constitution guarantees healthcare access for all, however inequalities still exist with regards to the burden of both communicable and non-communicable disease [2] as well as health access and funding distributions: almost 50% of total health expenditure is spent on 16% of the population covered by private medical insurance schemes [3]. The remainder is spent on the 84% of the population who depend on the under-resourced public sector, lack the means to access care in the private sector and have a higher disease burden [3, 4].

To address these inequities, the South African Government is in the process of rolling out a phased implementation of the NHI scheme over 15 years which will fund access for all for a specified basket of services at non-specialist level private health practitioners as well as public primary healthcare (PHC) facilities from a combined resource pool, thus expanding access to quality health services for those unable to afford these services while mitigating the financial burden on individuals and their families [3–5]. The proposed NHI aims to re-engineer primary healthcare, envisioning both private sector providers and public sector clinics to act as independent contracting units to the NHI Fund [3]. The goal is to create an integrated health-system which allows patients to access quality health services irrespective of socio-economic status [4]. Therefore apart from reducing financial barriers associated with the cost of accessing health services, the availability of health insurance will have an impact on health care seeking behaviour by influencing whether, when and from where care is sought [6]. It is estimated that 28% of South African households’ made use of the private sector as their primary access point to health care in 2016 [7]. In particular, it was estimated in 2006 that of those who are uninsured and with a household income less than $414 a month, approximately 22.4% of their most recent outpatient visits were to private general practitioners (GPs) [8]. In recent years, a number of private organisations in South Africa have established innovative models of PHC delivery that aim to provide access to good quality PHC services at affordable rates to the low-income, mostly uninsured and underserved population.

Understanding the factors which influence health seeking behaviour and service utilization within the public and private sector for this low income population has important implications for planning and contracting of services under the NHI due to potential shifts in volumes to the private sector. A recent South African study found socio-demographic and economic factors such as age, sex, education, employment and income to have a possible influence on health-care utilization [2]. Another study found that utilization is predicted by gender, perceived financial situation, mental and physical health, extra-household resources and the price of a private consultation while the number of visits is predicted by age, physical and mental health, extra-household resources and private provider quality [9]. There are however a limited number of studies which describe the health-seeking behavior of the low income predominantly uninsured population in detail and information gaps persist around factors influencing health access, utilization rates and out of pocket payments in the general South African population [2, 10]. There are also very few studies which comprehensively investigate health seeking behaviour exclusively from the patient perspective [11]. It is against this background that our study intended to provide a comprehensive description of healthcare seeking behaviour and service utilization as well as an understanding of the factors which influence this, from the patient perspective, within the low-income population accessing PHC services in either the public, private or a mix of both sectors. This study forms part of a larger analysis to assess the cost and outcomes of models of private PHC providers targeting this population relative to PHC service delivery at public sector clinics in South Africa.

## Methods

### Sampling

The population for this study included all patients, aged 18 years and older (adults), who presented for PHC services at seven selected private PHC organisations (primarily providing services to the low-income predominantly uninsured patient population) and their matched public sector clinic counterpart in the same, or adjacent clinic catchment area. Inclusion criteria for the matched private sector site was based on: Patient volumes across the chosen subset of PHC services and proximity to a public sector clinic, as previously described [12]. Only patients able to communicate in English, willing to provide written informed consent, and be physically present for an interview during our site visit were eligible for inclusion in the final random study sample. In order to achieve a representative sample with a precise confidence interval (10%) at alpha of 0.05 and power of 80%, a minimum sample size of 86 is needed, or 43 in total per sector. Assuming a maximum of 15 private PHC service delivery organisations interviewed for key informant interviews [12], and a matched sample of public sector PHC clinics, as well as 11 patient interviews per site. Participants were randomly selected through a systematic random sampling technique. Both the sampling framework and sampling interval used in this study were informed by expected daily volumes, as obtained through a discussion with relevant facility personnel.

### Data collection

This study was approved by the Human Research Ethics Committee of the University of Witwatersrand (ref. no. M171082) and the Institutional Review Board of the Boston Medical Centre (ref. no. H-37230). The study design was cross-sectional with data collected during once-off patient interviews. Face-to-face interviews were conducted with enrolled participants and an interviewer administered a semi-structured questionnaire. Interviews were conducted in a private room within the clinic to ensure confidentiality. Study data were collected and managed using the Research Electronic Data Capture (REDCap) tool; REDCap is a secure, web-based software platform designed to support data capture for research studies [13]. Patient interviews took place between May 2018 and January 2019. The interview guide was informed by the Quality of Life Survey (QoL) which is conducted and published by the Gauteng City- Region Observatory (GCRO) and measures a wide range of variables relevant to the purpose of our study including socio-demographic variables, health-seeking behaviour and attitudes towards the health-care services [14]. Data was collected on demographic characteristics, socio-economic indicators, the associated costs of accessing PHC over the last 12 months, healthcare services utilised over the last year, specifics around any chronic (HIV, TB, diabetes and hypertension) and health care requirements over the last year. Details on patient satisfaction with the care received were only collected for repeat patients to a facility All data collection methods were performed in accordance with the relevant ethics guidelines and regulations.

### Analysis

The selection of demographic, socio-economic and health related variables for this analysis was informed by the behavioural model for health service utilisation [15]. This model describes health service utilization as being conditional on three sets of factors: predisposing (demographic and social) factors, enabling (economic) factors, and need (health outcome) factors [16]. The outcome variable for this analysis was the PHC at which the participant indicated usually accessing care (public PHC, private PHC or mix of public and private facilities). Descriptive statistics were used to provide a summary of the study population across the usual PHC options. A chi-squared test was used to provide a crude test of associations between the PHC facility where care was usually accessed and the demographic, socio-economic and health related categorical variables of our study population. A one-way ANOVA test was used to identify any significant differences between the PHC facility where care was usually accessed and the continuous variables age, as well as household size. Healthcare utilisation over 12 months was determined for each option indicating where care was usually accessed based on responses to a question regarding the patient’s average number of health care visits per year. Response categories included: (i) More than once a month (weighted as 12 or more visits a year for this analysis), (ii) Once a month (12 visits a year), (iii) Once every 2 months (six visits a year), (iv) Four visits a year, (v) Three visits a year, (vi) Two visits a year, (vii) Once a year or (viii) Never. The frequency of participants falling into each category was then multiplied by the associated weighting in terms of annual visits per year and divided by the N for each option of where care is usually accessed to obtain the final healthcare utilisation value over 12 months. The influence of socio-economic status (SES) on choice of usual PHC was examined through the creation of an SES index, constructed from household and individual level data using principal component analysis [17]. Patients were then stratified into 3 equal sized groups, according to their SES level (low, medium and high) to identify any association between where care was usually accessed and SES level. All statistical data analysis was performed using Stata Statistical Software (Release 15. College Station, TX: StataCorp LLC). Income and costs are reported in 2019 USD.

## Results

### Sample characteristics

We enrolled and interviewed 153 participants across seven public and seven private PHC facilities – an average of 11 per study site. There was a shortfall of one participant at one study site. Table 1 shows the demographic, socio-economic and health related characteristics of our study sample. Two thirds (67%) of our study sample were female; the mean age was 40. The majority of our study population were economically active (63%), had no health insurance (91%) and earned up to $828 per month (71%). Almost two thirds (64%) reported having an educational level between Grade 8–12 (secondary/high school), 27% had completed their secondary/ high school education, while 3% had no formal education. The estimated mean household size was 4. The majority (58%) of our study participants indicated having either HIV, diabetes or hypertension or a combination of these diseases. Only 8% of participants indicated a willingness to switch the sector in which care (chronic or acute) was usually accessed.

**Table 1:** Demographic, socio-economic and health related characteristics of respondents, *N* = 153

Characteristics	N (%)
Gender, *n (%)*	
Male	51 (33)
Female	102 (67)
Age (mean, SE)	40 (1.05)
Relationship status, *n (%)*	
Married	43 (28)
Unmarried	110 (72)
Education, *n (%)*	
No Education	4 (3)
Some primary education (Grade (GR) R – GR 6)	15 (10)
Completed primary education (GR 7)	8 (5)
Some secondary education (GR 8-GR11)	56 (37)
Completed secondary education (Gr12/matric)	41 (27)
Certificate/diploma from college/technical college /university	23 (15)
Undergraduate from college/ technical college /university	6 (4)
Health insurance, *n (%)*	
No	139 (91)
Yes	14 (9)
Monthly income, *n (%)*	
≤ $69	18 (12)
$69.01 - $414	70 (46)
$414.01 - $828	20 (13)
$828.01 - $1724	6 (5)
> $1724	1 (1)
Refused to specify/did not know	38 (25)
Household size (Mean, SE)	4 (0.18)
Economically active^a^, *n (%)*	96 (63)
Socio-economic status group, *n (%)*	
1 – low SES	51 (33)
2 – medium SES	51 (33)
3 – high SES	51 (33)
Willing to switch sector in which care is usually accessed	12 (8)
Chronic disease (HIV, diabetes, hypertension), *n (%)*	
No	65 (42)
Yes	88 (58)

^a^ A participant was classified as economically active if they reported working in either the formal sector, informal sector or being self-employed

When asked about the health sector where they usually accessed care, 73 (48%) indicated usually visiting a public health facility, 49 (32%) usually chose a private health facility and 31 (20%) frequently utilised a mix of both facility types (Table 2). Education, health insurance being economically active and SES were significantly associated with usual PHC choice (*p* ≤ 0.05).

**Table 2:** Demographic, socio-economic and health related characteristics of the study population by usual PHC choice

Characteristic	PHC usually accessed (usual PHC)						***p***-value^**a**^
	Public sector [***N*** = 73]		Private sector [***N*** = 49]		Mix of both sectors [***N*** = 31]		
	N (%)	95% CI	N (%)	95% CI	N (%)	95% CI	
Gender							0.11
Male	20 (27)	18–39	22 (45)	32–59	9 (29)	16–47	
Female	53 (73)	61–82	27 (55)	41–68	22 (71)	53–84	
Age (mean, SE)	39 (1.6)	35–42	42 (2)	38–46	40 (1.8)	36–43	0.44
Relationship status							0.12
Married	15 (21)	13–31	16 (33)	21–47	12 (39)	23–57	
Unmarried	58 (79)	69–87	33 (67)	53–79	19 (61)	43–77	
Education							0.02
No Education	3 (4)	1–12	1 (2)	0.3–13	0 (0)		
GR R – GR 6	7 (10)	5–19	4 (8)	3–20	4 (13)	5–30	
GR 7	5 (7)	3–16	2 (4)	1–15	1 (3)	0.4–20	
GR8-GR11	23 (32)	22–43	17 (35)	23–49	16 (52)	34–68	
Gr12/matric	27 (37)	26–49	9 (18)	10–32	5 (16)	7–34	
Certificate/diploma	8 (11)	6–21	10 (20)	11–34	5 (16)	7–34	
Undergraduate	0 (0)		6 (12)	6–25	0 (0)		
Health insurance							0.002
No	72 (99)	91–100	39 (80)	66–89	28 (90)	74–97	
Yes	1 (1)	0.2–9	10 (20)	11–34	3 (10)	3–26	
Monthly income ^b^							0.07
≤ $69	10 (14)	7–24	5 (10)	4–22	3 (10)	3–26	
$69.01 - $414	31 (42)	32–54	24 (49)	35–63	15 (48)	32–66	
$414.01 - $828	7 (10)	5–19	5 (10)	4–22	8 (26)	13–44	
$828.01 - $1724	0 (0)		5 (10)	4–22	1 (3)	0,4–20	
> $1724	0 (0)		0 (0)		1 (3)	0.4–20	
Not specified	25 (34)	24–46	10 (20)	11–34	3 (10)	3–26	
Household size (Mean, SE)	4 (0.29)		3 (0.28)		4 (0.38)		0.11
Economically active^c^	39 (53)	42–65	37 (76)	62–86	20 (65)	46–79	0.046
Socio-economic status group							0.02
1 – low SES	30 (41)	30–53	13 (27)	16–41	8 (26)	13–44	
2 – medium SES	28 (38)	28–50	12 (24)	14–38	11 (35)	21–54	
3 – high SES	15 (21)	13–31	24 (49)	35–63	12 (39)	23–57	
Willing to switch sector in which care is usually accessed	5 (7)	3–16	2 (4)	1–15	5 (16)	7–34	0.135
Chronic disease (HIV, diabetes, hypertension)							0.56
No	34 (47)	35–58	20 (41)	28–55	11 (35)	21–54	
Yes	39 (53)	42–65	29 (59)	45–72	20 (65)	46–79	

^a^*p*-value for a X^2^ test of association between the specified categorical population characteristic and usual PHC choice; *p*-values for the continuous variables age and household size are for a one-way anova test

^b^ The chi squared test on the monthly income variable did not include the 38 participants who refused to provide an income

^c^ A participant was classified as economically active if they reported working in the formal/informal sector or being self-employed

### Healthcare seeking behaviour and utilisation

**Fig. 1:**
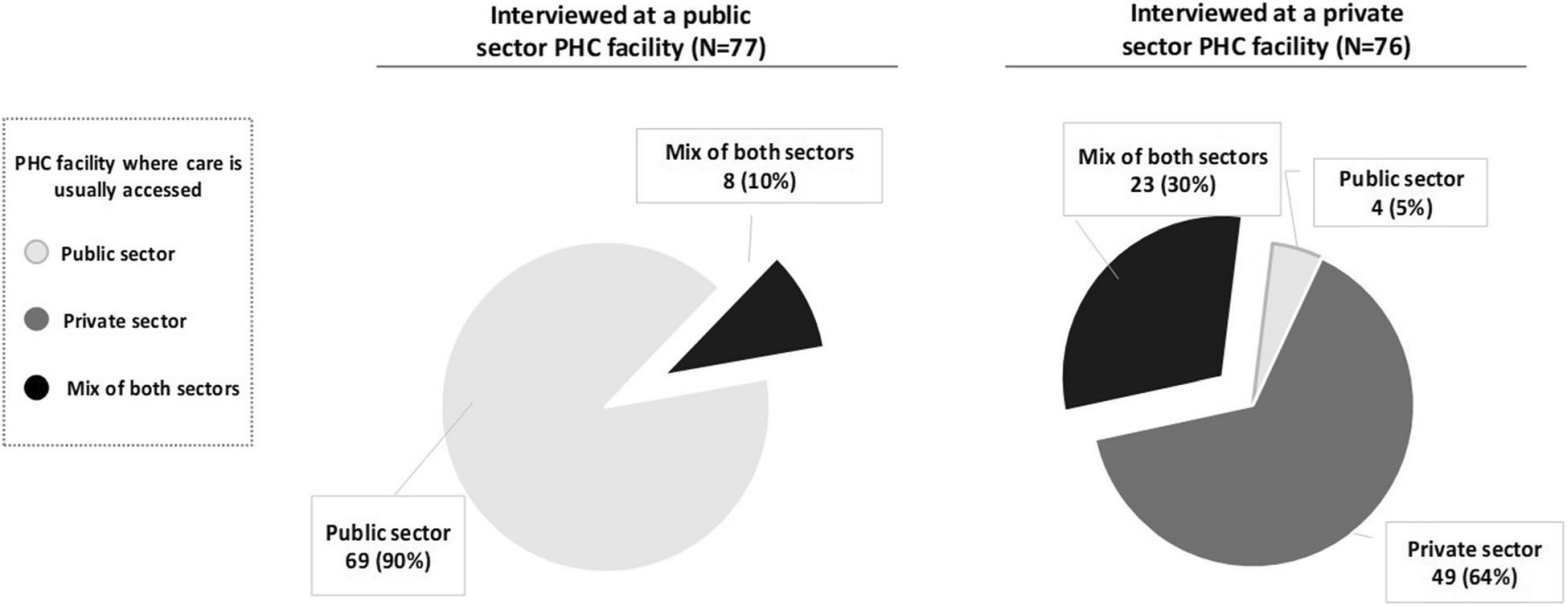
Usual PHC choice relative to the sector where the participant was interviewed

The majority of participants interviewed at both public and private facilities (84 and 82%, respectively) reported having accessed primary healthcare at that facility prior to enrolment. When asked to indicate the PHC clinic at which they usually access care, participants revealed a tendency to cycle between sectors. The proportion of participants using a mix of facility types was three times larger in the private sector than in the public sector (30% versus 10% respectively, Fig. 1).

**Fig. 2:**
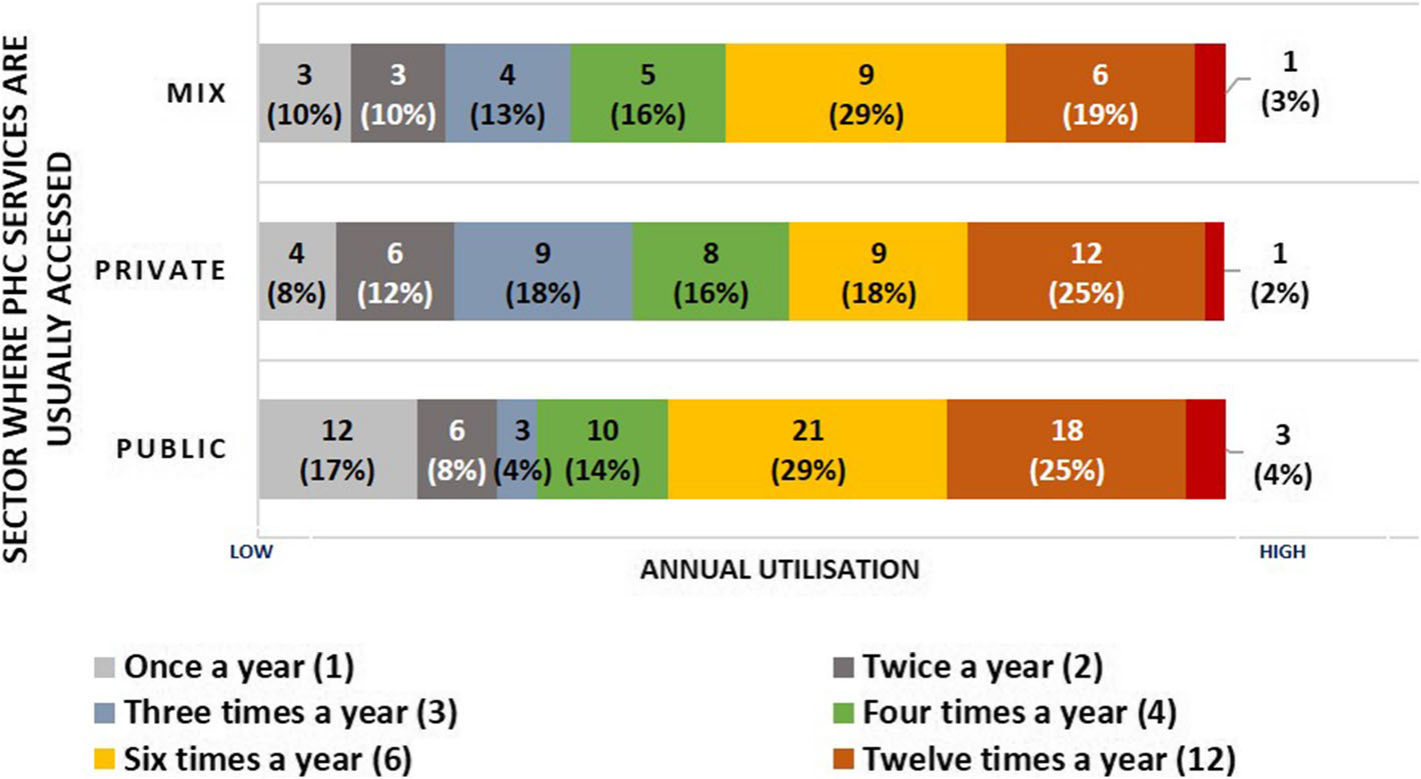
A comparison of healthcare utilisation for our study sample split by usual PHC choice

Healthcare utilisation over 12 months was similar across sectors, with a weighted average number of annual visits of 6.25 for those usually accessing care in the public sector (*N* = 73), 5.83 for those usually accessing care in the private sector (*N* = 49), and 5.81 for those using a mix of facilities (*N* = 31). An analysis of the utilisation distribution did however reveal a difference across the “Usual PHC” options (Fig. 2). The data showed higher visit frequency (defined as consulting a healthcare provider six or more times a year for this analysis) amongst those usually accessing care in the public sector (58% in comparison to 45% amongst those usually choosing to visit the private sector). The majority (80%) of participants with a higher visit frequency in our study sample had been diagnosed with one or more of the chronic conditions of interest in this research (i.e. HIV, diabetes or hypertension). Lower visit frequency (defined as consulting a healthcare provider three or fewer times a year for this analysis) was associated with usually accessing care in the private sector (38% in comparison to 29% in the public sector). These participants were also less likely to have been diagnosed with one or more of the chronic conditions of interest (38% reported being diagnosed with either HIV, diabetes, hypertension of a combination of these diseases).

**Fig. 3:**
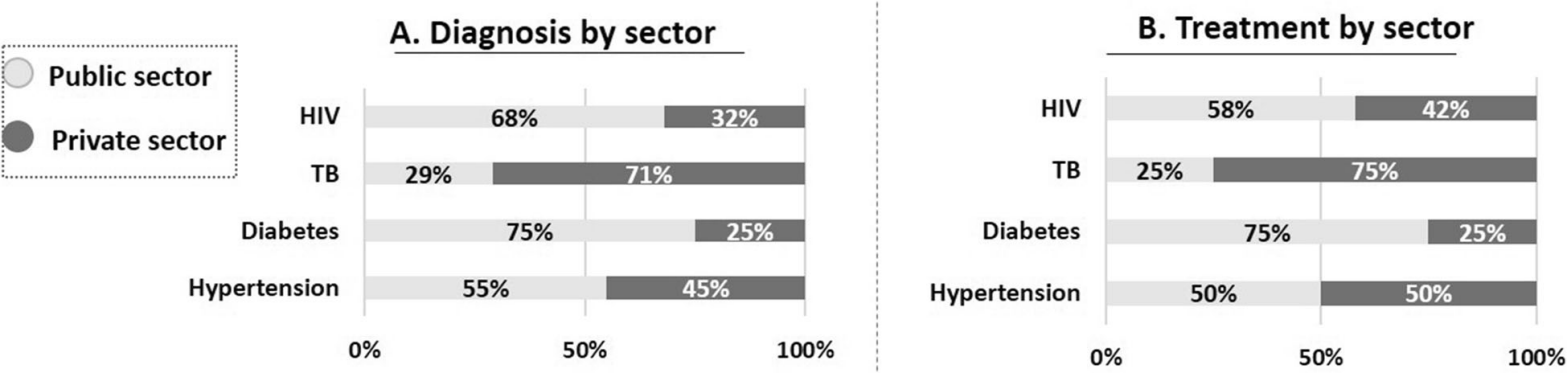
A comparison of diagnosis and treatment by sector

Whilst we interviewed an equal number of patients currently accessing private and public facilities, the type of facility where patients were diagnosed and treated for HIV, TB, diabetes and hypertension differed across sector (Fig. 3).

Equal numbers were diagnosed and treated for hypertension across sectors (50 and 45%), whereas more patients are diagnosed (75%) and treated (75%) for diabetes in the public sector. Despite the greater expense and specialised care required for TB and HIV, a relatively high proportion of TB and HIV patients are treated in the private sector (75 and 42% respectively). More patients were diagnosed (71%) and treated (75%) for TB in the private sector, but this is probably driven by the low numbers (5/7) were diagnosed with TB in the private sector and 3/4 were treated for TB in the private sector) and the inclusion of a PHC clinic with certain specialised TB services in the private sector sample.

Patients were asked to list the different healthcare services that they had accessed over the last 12 months and where they access them. They could choose more than one service, e.g. chronic services (related to diabetes, hypertension, HIV, TB, asthma, cancer etc.) and/or acute services (including minor ailments), and/or ‘other’ services including maternal and child health, sexual and reproductive health services, and support services (eye, oral, palliative etc.) (Table 3). An analysis of all services and facilities accessed by patients over the last 12 months revealed that acute services and chronic care services were the most commonly accessed services (46 and 43%). The majority of public hospital and private clinic visits were for acute services (64 and 57%), whilst the majority of public PHC clinics and private GP visitswere for chronic care services (44 and 52%). Our sample of patients only accessed private hospitals twice in the preceding 12 months: one for acute services and one for maternal and child health services. Public clinics provide the majority of maternal and child health care services (included in the ‘Other’ category).

**Table 3:** All healthcare services accessed over the last 12 months by sector and facility type

Service and facility classification		Service area			
		Chronic care	Acute care	Other	Total
		N (%)	N (%)	N (%)	N (%)
**Primary care services**	Public clinic	41 (44)	34 (36)	19 (20)	94 (47)
	Private clinic	20 (43)	27 (57)	0 (0)	47 (24)
	GP	14 (52)	12 (44)	1 (4)	27 (14)
**Secondary/tertiary services**	Public hospital	10 (36)	18 (64)	0 (0)	28 (14)
	Private hospital	0 (0)	1 (50)	1 (50)	2 (1)
		85 (43)	92 (46)	21 (11)	198 (100)

### Drivers of usual PHC facility choice

Answers to multiple response questions indicated that affordability (67%) and a convenient location (60%) were the main reasons for usually accessing care at a public health facility. Those participants who usually accessed care in the private sector specified convenience of location (71%) and quality of the care received (59%) as their main reasons behind facility choice. Finally, participants usually utilising a mix of facilities indicated similar key reasons for visiting each sector type, a convenient location (48%) and affordability (39%) were the main drivers of public sector access while quality of care (71%) and location convenience (42%) emerged as key drivers of private sector access for this segment of the study population. Refer to Table 4 for more detail.

**Table 4:** Reasons for usually using a public or private facility or mix of both (this was a multiple response question)

PHC usually accessed (usual PHC)	Reason for usually using public facilities		Reason for usually using private facilities	
	Public Sector (***N*** = 73)	Mix of both sectors [***N*** = 31]	Private Sector (***N*** = 49)	Mix of both sectors [***N*** = 31]
	N (%)	N (%)	N (%)	N (%)
**It is affordable**	49 (67)	12 (39)	10 (20)	0 (0)
**I receive good quality of care**	9 (12)	4 (13)	29 (59)	22 (71)
**It is convenient for me to access**	44 (60)	15 (48)	35 (71)	13 (42)
**There are no private/public health care facilities close by**	0 (0)	0 (0)	0 (0)	0 (0)
**I have been before and they could help**	3 (4)	4 (13)	2 (4)	3 (10)
**The staff are friendly/helpful**	5 (7)	2 (6)	7 (14)	5 (16)
**It has the medicine I need**	12 (16)	8 (26)	5 (10)	9 (29)
**Other**^a^	0 (0)	2 (6)	2 (4)	1 (3)

^a^Other reasons specified included “It’s the first time I come to this facility”, “Confidentiality is guaranteed, “Do not use public facilities” and “I had an acute condition”

### Patient satisfaction

All repeat patients to the facility of interview were asked to describe their level of satisfaction with the care received at that clinic: 97% of private sector clients were either very satisfied or satisfied with the service they received as compared to 74% in the public sector. The public sector had a higher proportion of patients who were either dissatisfied or indifferent (neither satisfied/ dissatisfied). This was reflected in the open-ended comments patients were asked to provide on their experience of care: 73% of negative comments (i.e. slow or long waiting times, staff shortages, inadequate opening hours or cramped facilities etc.) were by patients who attended a public sector clinic, whilst 63% of positive comments on the experience of care (friendly and good staff etc.) were by patients attending a private sector clinic (Table 5).

**Table 5:** General comment on experience of care by sector

Experience of care		Public sector	Private sector	Total
		N (%)	N (%)	N (%)
**Negative**	Slow/long waiting times	11 (85)	2 (15)	13 (100)
	Hours/size^a^	3 (75)	1 (25)	4 (100)
	Other	6 (55)	5 (46)	11 (100)
	Staff shortage	4 (80)	1 (20)	5 (100)
**Positive**	Satisfied	10 (37)	17 (63)	27 (100)
	Friendly/good staff	1 (17)	5 (83)	6 (100)
	Other	3 (60)	2 (40)	5 (100)
**Total**		38 (54)	33 (47)	71 (100)

^a^Includes comments referring to inconvenient opening hours or cramped facilities

## Discussion

This study has provided a description of health seeking behaviour amongst the low-income predominantly uninsured population who choose to access PHC services at either a public facility, private facility or utilise a mix of facility types in 2 provinces in South Africa. Results indicate a willingness to cycle between sectors; this movement is currently most likely constrained by finances and socio-economic status.

The perceived benefits and quality of care reported by participants is most likely a strong contributing factor to movement of patients between the public and private sector when health care is deemed urgent or critical and funds make this possible. These findings align with those reported by a previous study which found that patients in Ghana choose to access care in the private sector when financially viable, and preferred these facilities over government facilities covered by the Ghanaian NHI, mostly due to their perception of better quality of care in the private sector [18]. Another study looking at private providers operating under NHI schemes in both Ghana and Kenya found that study participants from both countries expressed an overall preference for accessing care at NHI accredited private sector facilities, with most citing shorter waiting times and more respectful treatment as the reasons for this; these participants also felt that NHI coverage not only provided greater access to healthcare, but also allowed them to access higher quality private clinics which they preferred over public sector facilities [19]. Similarly a previous South African study found that private health care played an important role in the health care decisions of poor South Africans who indicated a preference for the private sector when affordability constraints allowed [20]. A discrete choice experiment (DCE) undertaken in the Western and Eastern Cape provinces of South Africa also revealed a preference to not to seek care at a public facility, with the probability of attending public health facilities strongly influenced by attributes related to clinical quality such as the availability of medication [21]. Access to additional funds and perceived quality of service from a private provider influence where healthcare is accessed [9].

Finally a population-based study of the healthcare seeking behaviour of adults in Burkina Faso found that the utilization of private for profit health facilities has been shown in previous research to be dependent on factors such as insurance coverage, high education level, and being a formal job holder [22]. These findings are consistent with our results which similarly indicate that education, health-insurance, and socio-economic status are strong predictors of where care is usually accessed. Previous studies in low income countries have also shown that education influenced choice of providers [23, 24]. Furthermore, better educated and wealthier participants in a Ghanaian based study were significantly more likely to visit private health facilities compared to public health facilities [24]. Importantly, given the context of this current study, controlling for health-insurance was previously found to lessen the influence of factors such as education and occupation [22]. Our study reveals that potential impact on volumes could mean that while most high visit frequency patients currently access care in the public sector, a portion of these visits may be distributed to the private sector under NHI which might result in more than the estimated three annual visits per person per year [25, 26]. Further research on a larger sample is needed to confirm the robustness of these findings and to explore the influence of select population characteristics and UHC on health seeking behaviour within this low-income largely uninsured population.

This study helps provide a better understanding of healthcare utilisation in South Africa amongst the low-income population and shows potential implications on health-seeking behaviour for the implementation of NHI. Its limitations include the small study sample, future studies into the topic should consider assessing predictors from a very large population to maximize the power to detect significance and further analyse predictors reliably.. The study also asked for sensitive information such as monthly income which may be subject to over/under-reporting bias, as such this gives rise to another study limitation where 25% of our sample refused to report their monthly income which can bias the reported estimates. Another study limitation is the inherent selection bias in including patients who choose to use these PHC services and excludes the viewpoint of those who do not currently access services but may choose to do so under NHI, future research should consider addressing this shortcoming through at the population level through a population based survey.

This is a cross-sectional study therefore causal relations could not be ascertained. Lastly, while data on health seeking behaviour for a 12-month period was obtained during interviews, this information could have been subject to recall and response bias.

## Conclusions

The results of our study indicate cycling between the private and public sectors, with patients willing to seek care in the private sector when financial constraints allow. Access to this sector is currently most likely limited by finances and socio-economic status. With the implementation of NHI focused on removing financial barriers and enabling access to quality healthcare regardless of socio-economic group, affordability should no longer be a constraint on accessing care outside of the public sector. Understanding the potential impact on healthcare utilisation once affordability is mitigated through the NHI is important for planning, and has implications for the set-up of contracting systems for services under the NHI.

## Data Availability

The datasets supporting the conclusions of this article are available upon request via email from the authors.

## Abbreviations

DCE: Discrete choice experiment
NHI: National health insurance
PHC: Primary healthcare
REDCap: Research Electronic Data Capture
SES: Socio-economic status
UHC: Universal health coverage
